# New pathobiochemical insights into dystrophinopathy from the proteomics of senescent *mdx* mouse muscle

**DOI:** 10.3389/fnagi.2014.00109

**Published:** 2014-06-03

**Authors:** Ashling Holland, Paul Dowling, Kay Ohlendieck

**Affiliations:** Department of Biology, National University of IrelandMaynooth, Ireland

**Keywords:** aging, dystrophin, dystrophin-glycoprotein complex, dystrophinopathy, mass spectrometry, muscle aging, proteomics, sarcopenia

## Abstract

Primary abnormalities in the dystrophin gene cause X-linked muscular dystrophy, a highly progressive muscle wasting disorder of childhood. A spontaneous animal model of Duchenne muscular dystrophy is the *mdx* mouse, which presents a highly interesting phenotype that exhibits considerable variations in the degree of fiber degeneration in different subtypes of muscles. The idea that aging exacerbates the dystrophic *mdx* phenotype, as previously indicated by a large number of biochemical and cell biological studies, was clearly confirmed by comparative muscle proteomics. Here we outline recent findings of age-dependent changes in the dystrophin-deficient muscle proteome and contrast these results with the previously established proteomic profile of sarcopenic muscle. Besides comparable perturbations of various biochemical functions, especially striking are similarities in the cellular stress response associated with a drastic up-regulation of small αB-crystallin-like heat shock proteins. Hence, the comparison of large-scale proteomic data sets of natural muscle aging with dystrophic sarcopenia promises to shed light on the differential effect of sarcopenia of old age vs. senescent abnormalities on a mutant dystrophic background.

## Introduction

A high degree of load bearing and the continuous strain of excitation-contraction-relaxation cycles exert considerable physical tension on the peripheral structures of muscle fibers. In conjunction with sarcolemmal integrin complexes and cytoskeletal spectrin networks, the dystrophin-glycoprotein complex presents a major stabilizing protein assembly that counter-acts potential contraction-induced rupturing of the muscle surface membrane (Gumerson and Michele, 2011). The integral glycoprotein β-dystroglycan forms the core of this plasmalemma-spanning complex by interacting with the dystrophin isoform Dp427 on the inside of muscle fibers and concurrent binding to its extracellular subunit α-dystroglycan on the outside of contractile cells (Ibraghimov-Beskrovnaya et al., 1992). Since α-dystroglycan is a receptor of the extracellular matrix protein laminin and dystrophin acts as a cytoskeletal actin-binding protein, this complex structure confers flexibility to the muscle periphery and also anchors signaling molecules and ion channels within the sarcolemma region. Additional core members of the dystrophin-associated protein complex are sarcoglycans, dystrobrevins, syntrophins, and sarcospan (Ohlendieck, 1996; Ervasti, 2007).

In Duchenne muscular dystrophy, primary genetic abnormalities in the dystrophin gene cause the loss of the full-length Dp427 isoform of this membrane cytoskeletal protein (Hoffman et al., 1987) and the drastic reduction in all dystrophin-associated glycoproteins (Ohlendieck et al., 1993). Destabilization of sarcolemmal integrity results in an increased susceptibility to membrane micro-rupturing and complex cellular dysregulations (Rahimov and Kunkel, 2013), which play a central role in calcium-dependent damage pathways in muscular dystrophy (Allen et al., 2010). In this Perspective Article, the age-related exacerbation of the dystrophic phenotype is discussed and new pathobiochemical insights into dystrophinopathy outlined as revealed from the proteomic profiling of senescent *mdx* mouse muscles.

## Pathophysiological suitability of the mdx mouse model of X-linked muscular dystrophy

The *mdx* mouse is an internationally established animal model of Duchenne muscular dystrophy that is characterized by (i) a point mutation within exon 23 of the dystrophin gene, (ii) loss of the Dp427 isoform due to the premature termination of the full-length dystrophin polypeptide chain, (iii) reduction in all dystrophin-associated glycoproteins, (iv) general muscle damage as indicated by elevated levels of serum creatine kinase, (v) a high susceptibility to osmotic shock, (vi) an increased vulnerability to contraction- or stretch-induced injury, (vii) an enhanced cellular stress response, (viii) impaired excitation-contraction coupling, (ix) a lowered calcium buffering capacity in the sarcoplasmic reticulum, and (x) a chronic cytosolic calcium overload affecting rates of proteolysis (Banks and Chamberlain, 2008). Although one has to take into account the limitations of animal models for studying complex human diseases (Partridge, 2013), the *mdx* mouse can be conveniently used for determining basic pathophysiological mechanisms of dystrophinopathy and testing new pharmacological strategies or gene therapeutic approaches. This makes the dystrophic *mdx* mouse model an essential part of the overall strategy to elucidate the molecular pathogenesis of X-linked muscular dystrophy and find novel treatment options to reverse muscle degeneration in dystrophin-deficient fibers (De Luca, 2012).

It should be noted that the absence of dystrophin does not result in the same downstream alterations in different subtypes of *mdx* muscles and aging clearly worsens the dystrophic phenotype. While limb muscles display segmental necrosis and moderate weakness, laryngeal and extraocular muscles exhibit minimal effects and the diaphragm is severely dystrophic and functionally impaired in the *mdx* mouse (Stedman et al., 1991). This makes the various subtypes of *mdx* muscles extremely interesting for studying secondary abnormalities in dystrophinopathies and determining the detailed molecular and cellular features of compensatory mechanisms. Importantly, because the aged phenotype of the *mdx* diaphragm and heart closely resemble the human pathology, senescent *mdx* tissues present ideal model systems to determine the underlying mechanisms of fiber alterations during progressive skeletal muscle degeneration and muscular dystrophy-associated cardiomyopathy.

## What proteome-wide effects are associated with a deficiency in dystrophin isoform DP427?

**Figure 1:**
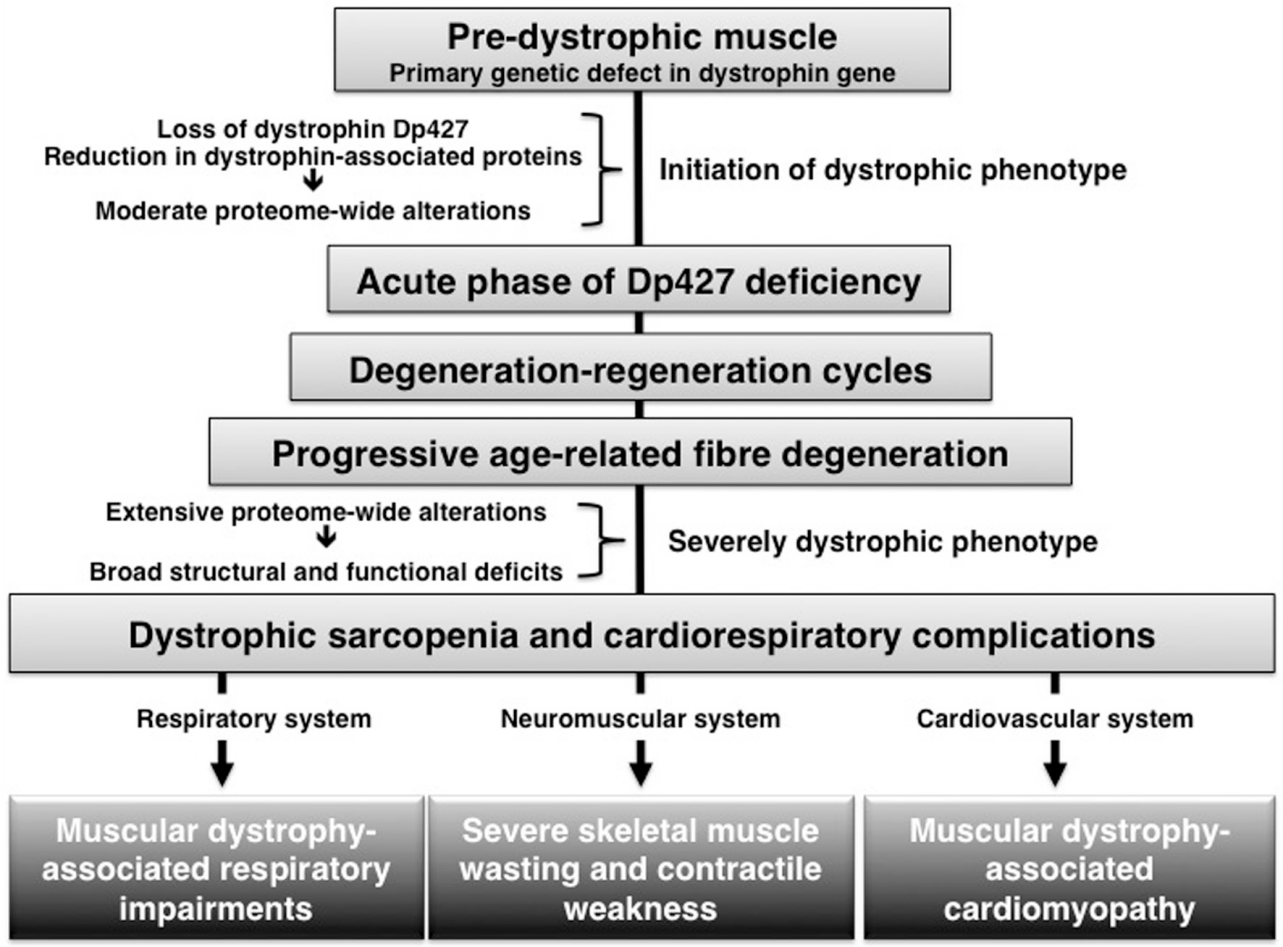
**Age-related progression of pathophysiological abnormalities in dystrophin-deficient muscle**.

The combination of large-scale protein separation techniques, such as two-dimensional gel electrophoresis and advanced liquid chromatography, and high-resolution mass spectrometry enable ultra-sensitive proteomic workflows (Altelaar and Heck, 2012). Over the last decade, mass spectrometry-based proteomics has been applied to studying the dystrophin-glycoprotein complex and the many downstream effects of dystrophin deficiency in muscular dystrophy (for review see, Holland et al., 2013a). Many of these investigations have focused on crude cellular extracts from the *mdx* mouse model of dystrophinopathy and the flow chart of Figure 1 outlines that the dystrophic *mdx* phenotype is characterized initially by moderate changes in the muscle tissue proteome, followed by considerably more severe proteome-wide changes in aged muscles on a mutant dystrophic background. The proteomic profiling of mildly dystrophic muscle subtypes revealed only very few changes in extraocular and *interosseus* muscles (Lewis and Ohlendieck, 2010; Carberry et al., 2013a). Segmental necrosis in moderately affected young *mdx* leg muscles was shown to be associated with changes in nucleotide metabolism (Ge et al., 2003) and generally perturbed muscle protein expression levels (Gardan-Salmon et al., 2011). Considerable changes in the degree and number of proteins was revealed by the fluorescence two-dimensional difference in-gel electrophoretic analysis of the adult *mdx* diaphragm muscle, which exhibits a variety of alterations in proteins involved in muscle contraction, ion homeostasis, nucleotide metabolism, the cellular stress response, energy metabolism and sarcolemmal signaling (Doran et al., 2006). Hence, dystrophin deficiency and the resulting collapse of the linkage between the intracellular actin cytoskeleton and the basal lamina triggers a variety of downstream modifications in muscular dystrophy.

## How does aging affect the dystrophic mdx phenotype?

The majority of proteomic surveys have focused on the acute phase of dystrophic changes in young *mdx* muscles or studied mature *mdx* muscle tissues. Recently, several proteomic investigations have also determined proteome-wide alterations during aging of the *mdx* model of dystrophinopathy. This is crucial, since aged *mdx* muscle exhibit pathological changes that more closely resemble the human pathology, including progressive contractile weakness due to the extensive loss of myofibers and replacement by connective and fatty tissue, abnormal signaling pathways, the appearance of branched fibers that trigger mechanical weakening of the sarcolemma, an impaired functional and structural recovery after injury and a drastic decline in regenerative potential (Holland et al., 2013a). The proteomic profiling of dystrophic sarcopenia has clearly demonstrated an exacerbated phenotype of muscle wasting.

The proteomic comparison of the senescent *mdx* diaphragm vs. age-matched wild type resulted in the mass spectrometric identification of 84 altered protein species. The new molecular insights into dystrophic changes in aged *mdx* mice indicated severely impaired calcium buffering, drastically elevated levels of cellular stress, cyto-structural alterations and metabolic disturbances in dystrophin-deficient muscle tissue (Carberry et al., 2013b). Although laminin is not affected in dystrophic skeletal muscles, aged cardiac *mdx* muscles showed a drastic reduction in laminin and nidogen (Holland et al., 2013b), which suggests a disintegration of the basal lamina structure and cytoskeletal network in cardiac fibers that lack the Dp427 isoform of dystrophin.

## How does the natural aging process affect the muscle proteome?

Biological aging is a multi-factorial process and associated with a large spectrum of physical ailments. The gradual loss of muscle mass and contractile strength increases the risk of poor balance, impaired mobility and frequent falling (Berger and Doherty, 2010). The systematic application of proteomics for studying aging has revealed muscle fiber transitions and metabolic shifts in senescent skeletal muscles (Piec et al., 2005; Gelfi et al., 2006; Gannon et al., 2009). Although mitochondrial impairments have been well documented to occur in aged human muscle (Théron et al., 2014) and animal models of sarcopenia (Ibebunjo et al., 2013), a glycolytic-to-oxidative shift is present in slower-twitching senescent muscles (O'Connell and Ohlendieck, 2009). These changes cannot be considered primary triggering factors of sarcopenia, but are most likely a pathophysiological consequence of changes in the peripheral nervous system and a higher susceptibility of fast vs. slow fibers to age-related degeneration processes (for review, see Ohlendieck, 2011). A recent quantitative analysis of age-associated changes in the mouse *gastrocnemius* muscle proteome revealed changes in key calcium-handling proteins (Hwang et al., 2014), which agrees with the idea of impaired excitation-contraction coupling and disturbed ion homeostasis in sarcopenia (O'Connell et al., 2008).

## Comparison of sarcopenia of old age vs. dystrophic sarcopenia

**Figure 2:**
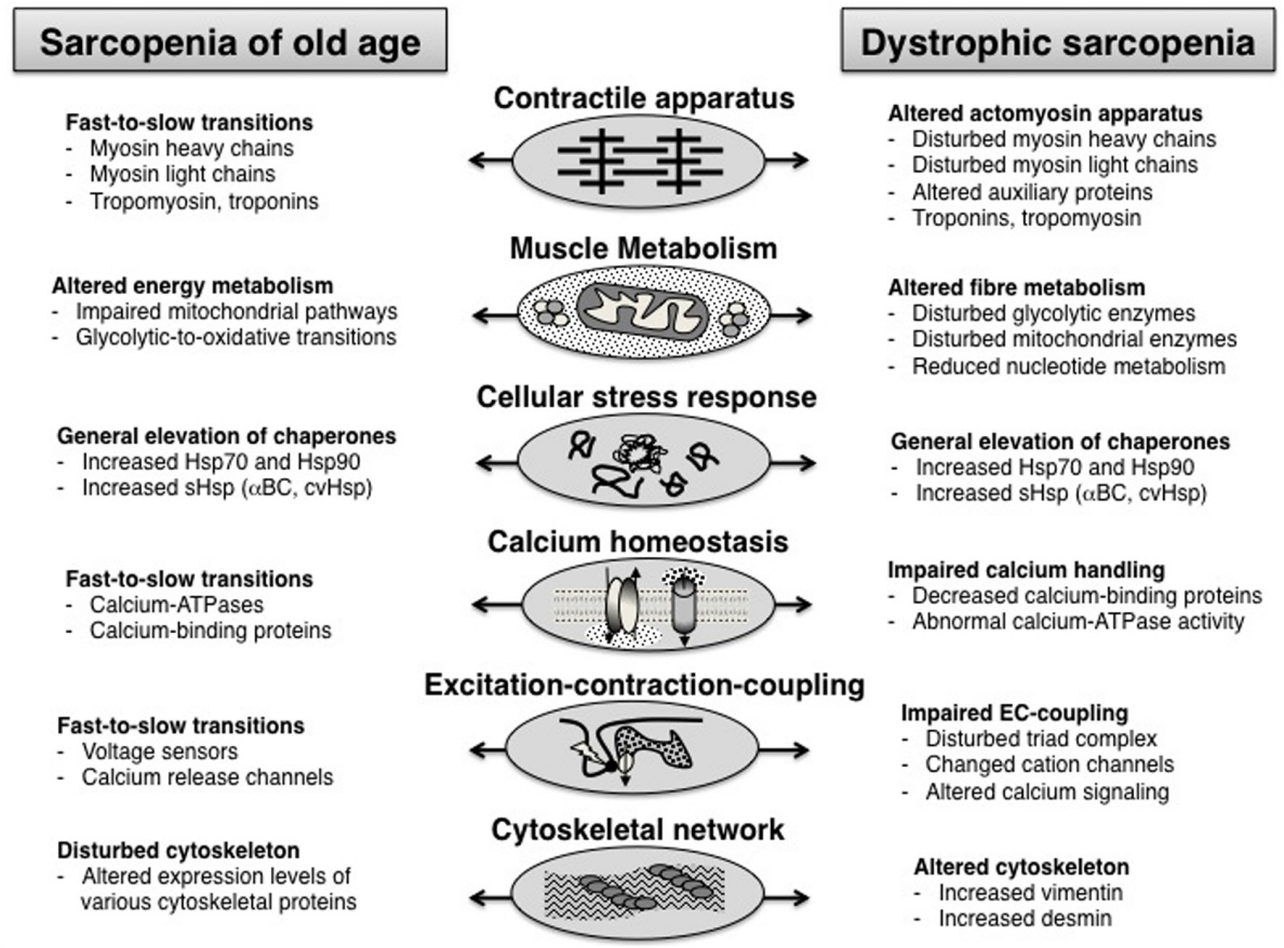
**Overview of proteome-wide alterations in the muscle tissue during sarcopenia of old age vs. dystrophic sarcopenia**.

Since both dystrophic sarcopenia and natural muscle senescence are characterized by a progressive loss of contractile tissue mass, elevated levels of fibrosis, a decline in the number of satellite cells, a drastically increased stress response and abnormal cellular signaling, it is interesting to determine whether these pathological similarities are reflected by analogous proteome-wide changes. The comparison of proteomic data sets from the analysis of muscular dystrophy vs. sarcopenia suggests that the pathobiochemical signature of certain damage or adaptation pathways is comparable, but that the molecular pathogenesis of both processes differs with respect to the degree of unilateral shifts in fiber types or energy metabolism. Both age-related processes show a disturbed abundance of proteins involved in excitation-contraction coupling, calcium homeostasis, cellular signaling cascades, the muscle contraction-relaxation cycle and the cellular stress response. For example, the expression of small heat shock proteins, such as αB-crystallin and some of its HSPB isoforms, is drastically increased in both natural muscle aging and dystrophic sarcopenia (Doran et al., 2006, 2007). Both types of skeletal muscle wasting are associated with altered levels of key enzymes involved in glycolysis, the citric acid cycle and oxidative phosphorylation (Piec et al., 2005; Doran et al., 2006, 2008). However, muscular dystrophy exhibits a generally perturbed abundance of metabolic enzymes, while the proteomic profiling of sarcopenia of old age clearly indicates a glycolytic-to-oxidative metabolic shift and concomitant fast-to-slow transformation on the level of the actomyosin apparatus (Ohlendieck, 2011). Figure 2 outlines the findings of the proteomic profiling of sarcopenia of old age vs. dystrophic sarcopenia.

## Conclusion

The application of proteomics for studying muscular dystrophy and aging has allowed the unbiased and hypothesis-generating analysis of the age-associated progression of the dystrophic phenotype. The combination of large-scale protein separation, high-throughput mass spectrometry and advanced bioinformatics has enabled the field of applied myology to evaluate global changes in muscle protein constellations. In both, cellular biogerontology and muscular dystrophy research, the identification of molecular changes in pathological tissues plays a crucial role in improving our detailed understanding of the fiber wasting process, and might also be helpful in refining diagnostic procedures, prognostic methodology and therapeutic approaches. Proteomic profiling has shown that the degree of change and the number of affected muscle proteins drastically increase in an age-related fashion in various subtypes of muscles in the dystrophic *mdx* mouse. This pathobiochemical exacerbation justifies the new term “dystrophic sarcopenia” for describing the age-related progressive phenotype of dystrophinopathy. It remains to be determined what exact role abnormalities in the nervous system play in dystrophinopathies and whether neuronal impairments increase with aging (Waite et al., 2012). In contrast to proteome-wide alterations during natural muscle aging, which is characterized by relatively unilateral metabolic and fiber type shifting, dystrophin-deficient fibers exhibit a more generally disturbed protein expression pattern during aging without distinct metabolic adaptations. Thus, sarcopenia appears to be more closely related to a differential susceptibility of individual fiber types to muscular atrophy, causing an overall fast-to-slow transition process, as compared to aged dystrophic muscle. Overall, the pathomechanisms of natural muscle aging and dystrophic sarcopenia share certain molecular and cellular modifications, but appear to differ in the fiber type specificity of their pathological susceptibility.

### Conflict of interest statement

The authors declare that the research was conducted in the absence of any commercial or financial relationships that could be construed as a potential conflict of interest.
